# Supplementary material for: Knowledge, attitude, and practice of Bangladeshi residents during COVID-19 pandemic
Source: PLOS Glob Public Health. 2022 May 9;2(5):e0000407. doi: 10.1371/journal.pgph.0000407 (PMC10021720; doi:10.1371/journal.pgph.0000407)
Supplement: S1 Questionnaire — (DOCX) [file pgph.0000407.s001.docx]

**Questionnaire Used for this Research:**

1. Your gender:

A. Man, B. Woman

2. Your age group:

A. 18-25, B. 26-49, C. 50+

3. Your place of residence:

A. Dhaka division, B. Outside Dhaka division

**KNOWLEDGE:**

K1. Are you confirmed about the differences between the symptoms of COVID-19 and the common flu?

1. Yes, B. No, C. Not sure

K2. Do you believe any of the proposed medicines could help to fight against COVID-19?

A. Yes, B. No, C. Not sure

K3. How do you maintain social distance from other people everywhere?

A. Keeping less than 1.5m distance, B. Keeping at least 1.5m distance, C. Not sure

K4. What is the most possible way to be infected in Bangladesh?

A. Getting close to someone infected, B. Touching things used by infected persons, C. Exposure to air

K5. What should you do after finding the COVID-19 symptoms in you?

A. Taking all preventive measures, B. Testing, C. hiding from others

**ATTITUDE:**

A1. How do you feel to see social attitudes and rudeness towards the COVID-19 positive patients?

A. Frustrated, B. Scared, C. Angry

A2. What risks do you predict after the lockdown is over?

A. Respreading, B. Normalize, C. Not sure

A3. What precautions might prevent you from infection after the lockdown?

A. Wearing safety masks/gloves, B. Maintaining social distance, C. Disinfecting hands and things frequently

**PRACTICE:**

P1. Which animal protein are you avoiding to keep safe from COVID-19?

A. Eggs and milk, B. Fish and meat, C. None

P2. How frequently do you go outside for any specific purposes like buying foods/medicines etc?

A. Once a day, B. Once a week, C. Staying home

P3. Do you disinfect your things and take a bath instantly after coming back from outside?

A. Always, B. Sometimes, C. Seldom

P4. What safety measures do you avail to prevent infection?

A. Drinking warm water, B. Taking steam, C. Avoiding cold foods and drinks

P5. What will you do initially if you are COVID-19 positive?

A. Homecare, B. Shifting to hospital, C. Not decided

P6. How do you raise positivity and awareness among others?

A. Using social media/group chat/online discussion, B. By practicing, C. By showing or demonstrating
