# Supplementary material for: Knowledge, attitude, and practice of Bangladeshi residents during COVID-19 pandemic
Source: PLOS Glob Public Health. 2022 May 9;2(5):e0000407. doi: 10.1371/journal.pgph.0000407 (PMC10021720; doi:10.1371/journal.pgph.0000407)
Supplement: Attachment — Submitted filename: Response to Reviewers.docx [file pgph.0000407.s002.docx]

Carl Abelardo T. Antonio

Academic Editor

PLOS Global Public Health

PGPH-D-21-00907

Title: Knowledge, Attitude, and Practice of Bangladeshi Residents during COVID-19 Pandemic

Submission deadline: Jan 13 2022 11:59PM

**Journal and Editorial Comments:**

1. PLOS ONE does not copy edit accepted manuscripts (https://journals.plos.org/globalpublichealth/s/criteria-for-publication#loc-5). To that effect, please ensure that your submission is free of typos and grammatical errors. *Please change "female” or "male" to "woman” or "man" as appropriate, when used as a noun (see for instance <https://apastyle.apa.org/style-grammar-guidelines/bias-free-language/gender>).

Answer: We updated this in the revised version.

2. If you have no competing interests to declare, please state "The authors have declared that no competing interests exist"

Answer: We updated this in the revised version.

3. If you did not receive any funding for this study, please simply state: “The authors received no specific funding for this work.”

Answer: We updated this in the revised version.

4. I concur with your peers that further description and explication of the methods and results is required for this manuscript before it is even considered for publication. While the changes required are extensive, I do think that these can be completed within the turnaround time of the journal.

Answer: We made the major revision and updated the whole manuscript including the results and discussion section. All are highlighted in yellow. We will do more revisions if necessary.

5. Have the authors made all data underlying the findings in their manuscript fully available (please refer to the Data Availability Statement at the start of the manuscript PDF file)? The PLOS Data policy requires authors to make all data underlying the findings described in their manuscript fully available without restriction, with rare exception. The data should be provided as part of the manuscript or its supporting information, or deposited to a public repository. For example, in addition to summary statistics, the data points behind means, medians and variance measures should be available. If there are restrictions on publicly sharing data—e.g. participant privacy or use of data from a third party—those must be specified.

Answer: All the data are publicly available and the link is given in the revised manuscript.

**Reviewer #1:**

1. It should be checked by an editor for grammatical errors to improve its readability.

Answer: We checked the full manuscript again and checked the grammatical errors and tried to improve the readability.

2. In the abstract, the authors specified that they used logistic regression and correlation analysis in their study. However, the main text did not mention this in the data analysis section.

Answer: We added this in the data section and updated the revised manuscript.

3. The authors should explain the rationale for employing statistical tests in their study. In particular, what objectives necessitate the use of the tests they presented?

Answer: It is updated in the result section.

4. If logistic regression is necessary, specify the variable selection procedure they used to determine the final model.

Answer: It is updated in the regression analysis section under the result section.

5. Authors should mention whether the assumptions of the statistical tests were met and how were these tested.

Answer: It is updated in the result section.

6. Study design was not explicitly specified.

Answer: It is updated in the research design section.

7. Sample size computation was not described.

Answer: It is updated in the data collection section.

8. The section concerning data collection included some information that should be placed under the Results section.

Answer: We moved the details in the results section.

9. Tables must be reformatted to include only important statistics. For instance, t- and F-values are unnecessary since p-value and confidence interval estimates were already reported.

Answer: We revised all the data tables as suggested.

10. There are too many tables. Consider deleting or merging some of the tables.

Answer: We removed few tables because some of these tables are unnecessary.

11. Consider adding sub-sections to the results section to streamline its contents.

Answer: We added few subsections in the revised manuscript.

12. Too many abbreviations were used. I suggest that the KAP questions be explicitly stated in the tables instead of using abbreviations.

Answer: We presented KAP questions in the appendix. If we add these questions in the tables then it looks very clumsy.

**Reviewer #2:**

1. The ethics review and approval process was not clearly and adequately described in the manuscript.

Answer: Our survey study is followed the research guidance and protocol of the University of Dhaka, Dhaka-1000, Bangladesh. This research survey is approved by Chairman, Department of Mathematics, University of Dhaka and gave us verbal consent to continue the present research. We also informed each of the participants that all the personal or institutional information’s gathered in this study provided by the participants will remain confidential. And if they agree, they can participate in this research survey.

2. Please expound in the methodology section what statistical tests were used for which specific objective or outcome of the study.

Answer: Updated as suggested.

3. It is recommended that the results presented in Table 8 be discussed in the first part of the results section.

Answer: We moved this part at the beginning of the results section.

4. What are the cut-off values for the variables knowledge, attitudes and practices? (i.e. What is the cutoff score for knowledge to be considered as good?)

Answer: Mean score for each case is considered as cutoff score for the variables knowledge, attitudes and practices.

5. The tabular presentation of data needs to be revised because the way that it is presented is not clear (e.g. what do the column headers A, B, C mean?). Finally, the labels for the tables need to be revised and include more elements (e.g. variable, statistic, population, time period, sample size).

Answer: We updated all the tables. Here, A, B, and C indicate three possible options for each knowledge/attitude/practice questions. Questions are presented in the appendix.

6. What does the phrase in the results and discussion and conclusion sections "the old age group is the most alert group" mean?

Answer: Old age group means age 50 or over. This group is more vulnerable and higher infection rate. So, this group takes more precautions in order to prevent COVID-19 infection.

7. The discussion section did not introduce the salient findings of the study. Furthermore, the results of the study were not adequately discussed in the section. What do the results imply? How is it compared to other studies? Comparison of the current study's findings were briefly addressed not in the discussion section, but in the results section.

Answer: We tried to update it and please see the revised manuscript. We improved the discussion section as well.

8. What are the limitations of the study? Also, the recommendations section can still be reduced.

Answer: We reduced the recommendation section. Updated in the manuscript.

9. In the discussion section, studies conducted in Pakistan and India were referenced but no in-text citations were included.

Answer: Updated in the revised version.

10. The article needs proofreading to improve its readability. There are some parts of the manuscript were words seem to be randomly inserted (e.g. the word "inquires" in the first sentence under the research design subsection 'tools'; the words "p value" and "counter" in A3)

Answer: We checked the full manuscript again and checked the grammatical errors and tried to improve the readability.

11. The in-text citation and reference list do not follow the journal's recommended format (Vancouver)

Answer: Updated the reference section using Vancouver format.
